# Glucose-Regulated Protein 94 Modulates the Response of Osteosarcoma to Chemotherapy

**DOI:** 10.1155/2019/4569718

**Published:** 2019-01-03

**Authors:** Chien-Yu Huang, Po-Li Wei, Jin-Wun Wang, Precious Takondwa Makondi, Ming-Te Huang, Hsin-An Chen, Yu-Jia Chang

**Affiliations:** ^1^Department of Surgery, School of Medicine, College of Medicine, Taipei Medical University, Taipei, Taiwan; ^2^Division of General Surgery, Department of Surgery, Shuang Ho Hospital, Taipei Medical University, Taipei, Taiwan; ^3^Cancer Research Center and Translational Laboratory, Department of Medical Research, Taipei Medical University Hospital, Taipei Medical University, Taipei, Taiwan; ^4^Division of Colorectal Surgery, Department of Surgery, Taipei Medical University Hospital, Taipei Medical University, Taipei, Taiwan; ^5^Graduate Institute of Cancer Biology and Drug Discovery, Taipei Medical University, Taipei, Taiwan; ^6^Department of Orthopedics, Chiali Chi-Mei Medical Center, Chiali, Tainan, Taiwan; ^7^Graduate Institute of Clinical Medicine, College of Medicine, Taipei Medical University, Taipei, Taiwan; ^8^International PhD Program in Medicine, Taipei Medical University, Taipei, Taiwan

## Abstract

**Background:**

Osteosarcoma (OS) is the most common and most aggressive primary solid malignant bone tumor in children and young adults and has high rates of recurrence and metastasis. The endoplasmic reticulum (ER) stress pathway is important in regulating the chemo-responsiveness of cancer. However, the role of glucose-regulated protein 94 (GRP94) in regulating the response of OS to chemotherapy has never been explored.

**Methods:**

In this study, two OS cell lines, MG63 and 143B cells, were used to evaluate the mechanism by which GRP94 modulates the response of osteosarcoma to chemotherapy. GRP94-knockdown (GRP94-KD) OS cells were generated using short hairpin RNAs, and the response to chemotherapy was assessed using an MTT (3-(4,5-dimethylthiazol-2-yl)-2,5-diphenyltetrazolium bromide) assay. Cell apoptosis was quantified with propidium iodide (PI) staining and flow cytometry.

**Results:**

Silencing of GRP94 in MG63 and 143B cells did not influence the growth and migration of the cells, but reduced the colony formation. GRP94-KD OS cells were more resistant to paclitaxel, gemcitabine, and epirubicin treatments than cells transfected with the scrambled control, and more cells transfected with the scrambled control underwent apoptosis after paclitaxel, gemcitabine, and epirubicin treatments than GRP94-KD cells.

**Conclusions:**

Therefore, GRP94 silencing may increase the resistance of MG63 and 143B cells to paclitaxel, gemcitabine, and epirubicin treatments by inhibiting the induction of apoptosis. Thus, GRP94 may be a key biomarker for the chemotherapeutic response of OS.

## 1. Introduction

Osteosarcoma (OS) is the most common type of primary solid malignant bone tumor in children and young adults (nearly 5% of all cases of cancer in children), with 70–75% of cases occurring between the ages of 10 and 25 years [1]. OS forms at the ends of the long bones of the body, such as in the arms and in the legs, mainly near the knee [2]. OS is an aggressive disease with a high recurrence rate after treatment and is highly metastatic to lungs and bones, thus leading to a poor prognosis [1, 3]. Currently, the standard therapeutic strategy for OS is surgical resection and chemotherapy. However, although several new drugs have been developed in the past decade, the efficacy of these treatments is not satisfactory [3]. Moreover, the lack of prognostic biomarkers for early diagnosis and therapeutic responses is a major issue in the management of OS.

Stress-related proteins such as glucose-related protein 94 (GRP94) play critical roles in tumor progression and therapeutic efficacy. GRP94 has been shown to aid cells in evading lethal stresses, such as ischemic injury, radiation exposure, and chemotoxicity [4–6]. As a member of the heat shock protein 90 (HSP90) family of molecular chaperones, GRP94 is located in the ER. GRP94 is overexpressed in cancer tissues, and this overexpression is associated with cancer aggressiveness, the metastatic potential, and chemotherapy responses [7, 8]. Strategies targeting GRP94 have been shown to enhance the degradation of GRP94 client proteins and to induce cell apoptosis in different cancers [9, 10]. According to previous clinical trials, a GRP94-targeting drug reduces metastasis and improves the responses of specific cancers to chemotherapy [11–13].

The roles of GRP94 in the progression and therapeutic response of OS are not clear. Therefore, we aim to explore the roles of GRP94 in OS to ultimately determine effective approaches for managing the disease.

## 2. Materials and Methods

### 2.1. Chemicals, Reagents, and Cell Culture

Human OS cells MG63 were purchased from ATCC and cultured in Eagle's minimum essential medium (MEM) (Gibco BRL, Grand Island, NY, USA) containing 2 mM L-glutamine, 1.5 g/L sodium bicarbonate, 10% fetal calf serum (Gibco BRL, Grand Island, NY, USA), and 2% penicillin-streptomycin (10,000 U/mL penicillin and 10 mg/mL streptomycin). The 143B cell line was provided by Dr. Pei-Ni Chen (Chung-Shan Medical University) and cultured in RPMI supplemented with 10% fetal bovine serum. Cells were incubated in a humidified incubator (37°C, 5% CO_2_) and were either subcultured or used before they reached 80% confluence. Triton X-100, Tris-HCl, neomycin, trypan blue/EDTA, ribonuclease-A, and dimethyl sulfoxide (DMSO) were obtained from Sigma Chemical Co. (St. Louis, MO). Antibodies against GRP94 and GAPDH were purchased from Santa Cruz Biotechnology, Inc. (Santa Cruz, CA). Caspase 3, caspase 7, and PARP antibodies were purchased from Cell Signaling Technology (Danvers, MA, United States).

### 2.2. Generation of GRP94-Knockdown OS Cell Lines

The expression of GRP94 in MG63 cells and 143B cells was silenced using a small hairpin RNA (shRNA). A GRP94-specific shRNA was purchased from the National RNAi Core Facility, Academia Sinica, Taiwan, and was described in a previous study [14]. The target sequences for human GRP94 mRNA (NM_003299) and a nontarget shRNA were described in the same study. The GRP94-shRNA and control-shRNA plasmids were transfected into MG63 cells and 143B cells using a Neon® Transfection System (Life Technologies, Grand Island, NY). The stably transfected cells were selected using the antibiotic puromycin, as previously described [15, 16]. After 48 h, the expression of GRP94 was verified by quantitative real-time PCR and Western blotting.

### 2.3. Protein Extraction and Immunoblot Analysis

Protein abundance was determined using sodium dodecyl sulfate-polyacrylamide gel electrophoresis (SDS-PAGE) and immunoblotting, as previously described [17]. Cells were washed with cold PBS and lysed with cell lysis buffer containing protease inhibitors (Boehringer Mannheim, Indianapolis, IN). Equal amounts of proteins were separated on a 10% SDS-PAGE gel under reducing conditions and transferred onto PVDF membranes (Bio-Rad Laboratories, Hercules, CA). Membranes were subsequently blotted using antibodies against GRP94, caspase 3, caspase 7, PARP, or GAPDH and horseradish peroxidase-conjugated secondary antibodies, visualized using TOOLS Ultra ECL-HRP Substrate (BIOTOOLS Co., Ltd., Taiwan), and then detected using a VersaDoc 5000 imaging system (Bio-Rad Laboratories).

### 2.4. Cell Viability Assay

Cells were plated in 24-well plates at a density of 2 × 10^4^ cells/well and incubated overnight. Cells were incubated with different concentrations of paclitaxel (0–600 ng/mL), gemcitabine (0–40 *μ*g/mL), or epirubicin (0–2 *μ*g/mL) for various periods to determine the dose that resulted in 50% inhibition (IC_50_). dH_2_O was used as a vehicle control. The medium was aspirated at selected time points. The remaining cells were further incubated with 0.25 mg/mL MTT for 1 h and subsequently extracted with DMSO, and the color change in the extract was measured at 515 nm using a spectrophotometer (GE Healthcare).

### 2.5. DAPI Staining

Approximately 2 × 10^5^ cells/well in a four-well chamber slide were incubated with paclitaxel (6 ng/mL), epirubicin (2 ng/mL), or gemcitabine (4 ng/mL) for 48 h. The cells were then fixed, stained with 4′,6-diamidino-2-phenylindole (DAPI), and imaged using a fluorescence microscope.

### 2.6. Propidium Iodide (PI) Staining for Determining Apoptosis

Cells (3 × 10^5^) were seeded into six-well plates and allowed to adhere overnight. Cells were incubated with paclitaxel (6 ng/mL), gemcitabine (4 ng/mL), or epirubicin (2 ng/mL) for 48 h and then harvested and washed with PBS at different time intervals. Then, the cells were fixed with pure methanol, treated with RNase A at a final concentration of 40 *μ*g/mL, and stained with propidium iodide (40 *μ*g/mL) for 30 min at room temperature. The stained cells were analyzed using Attune NxT Flow Cytometer (Thermo Fisher Scientific, Waltham, MA, United States), and the DNA content was quantified using the Modfit software (Verity Software House, Inc., Topsham, ME). The percentage of hypodiploid cells (sub-G_1_) was used to quantify dead cells. The results were analyzed using FlowJo Software.

### 2.7. Transwell Migration Assay

*In vitro* cell migration was investigated using an 8 *μ*m BD Falcon™ culture insert (BD Biosciences), as previously described [18]. Specifically, five hundred cells were suspended in 500 *μ*L of serum-free media and then seeded into the upper compartment of the chamber. The lower compartment was filled with 1 mL of media containing 10% FCS. After 24 h of incubation, the nonmigrating cells were scrubbed from the upper surface of the membrane. The migrated cells on the reverse side of the membrane were stained with 0.1% crystal violet, and the cells were counted under a microscope at 100-fold magnification.

### 2.8. Colony Formation Assay

Cells were seeded in a 6-well plate at a density of 10000 cells/well and cultivated for 2 weeks. Subsequently, cells were fixed and stained with crystal violet. Crystal violet staining was observed and quantified under a phase contract microscope.

### 2.9. Analysis of Caspase 3/7 Activity

Caspase 3/7 activity was measured using a SensorLyte® Homogeneous AMC Caspase 3/7 assay kit, according to the manufacturer's instructions (AnaSpec, Inc., Fremont, CA). Fluorescence intensity was measured using a Varioskan Flash (Thermo Fisher Scientific, Waltham, MA) at an excitation wavelength of 354 nm and an emission wavelength of 442 nm.

### 2.10. Statistical Analysis

All experiments were repeated a minimum of three times. All data reported are presented as means ± SD. The data presented in the figures were obtained from representative experiments and were quantitatively similar to the replicate experiments. Statistical significance of differences in data between two samples was determined using Student's *t*-test (two-tailed) with Microsoft Excel.

## 3. Results

### 3.1. GRP94 Silencing Did Not Influence the Proliferation or Migration but Reduced the Colony Formation Ability of OS Cells

**Figure 1:**
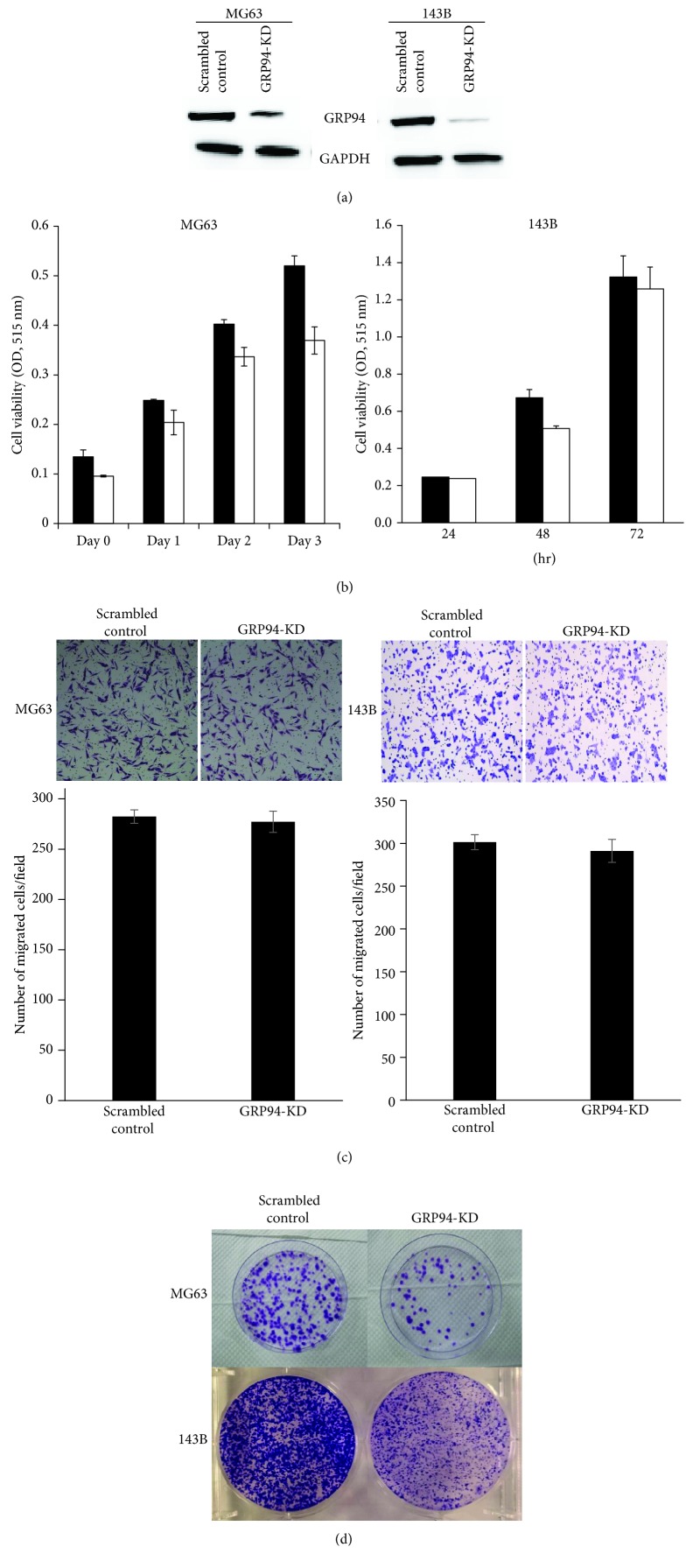
The effects of downregulating GRP94 expression on MG63 and 143B cells. (a) GRP94 was silenced using an shRNA. The levels of GRP94 in the control and GRP94-knockdown (GRP94-KD) MG63 and 143B cells were confirmed by Western blotting. (b) The growth of GRP94-KD and scrambled control MG63 and 143B cells was determined using the MTT assay. (c) Migration was assessed using transwell migration assays. (d) The colony formation assay was performed using scrambled control and GRP94-KD cells. The results reported were obtained from at least three independently repeated experiments (^∗∗^*p* < 0.01).

GRP94 expression was knocked down with an shRNA, and stably transfected cells were selected using antibiotics to further dissect the role of GRP94 in OS. The knockdown efficiency was confirmed by Western blotting, and GRP94 expression in knockdown cells was reduced by greater than 80% at both the transcriptional and translational levels compared with that in cells transfected with the scrambled control (Figure 1(a)). The growth of GRP94-KD and scrambled control MG63 cells was determined using the MTT assay to analyze the biological effects of the downregulation of GRP94 expression on MG63 cells and in 143B cells. As shown in Figure 1(b), the growth of GRP94-KD cells and scrambled control OS cells was similar. Moreover, the transwell migration assay did not reveal differences in the number of migrating cells between the GRP94-KD and scrambled control MG63 and 143B cells (Figure 1(c)). Interestingly, the numbers of colonies were dramatically reduced in GRP94-KD cells compared with scrambled control cells (Figure 1(d)). These results obtained after GRP94 silencing suggest that GRP94 does not influence the growth or migration of MG63 and 143B cells but may mediate the ability of OS cells to form colonies.

### 3.2. Knockdown of GRP94 Increased the Resistance of OS Cells to Chemotherapy

**Figure 2:**
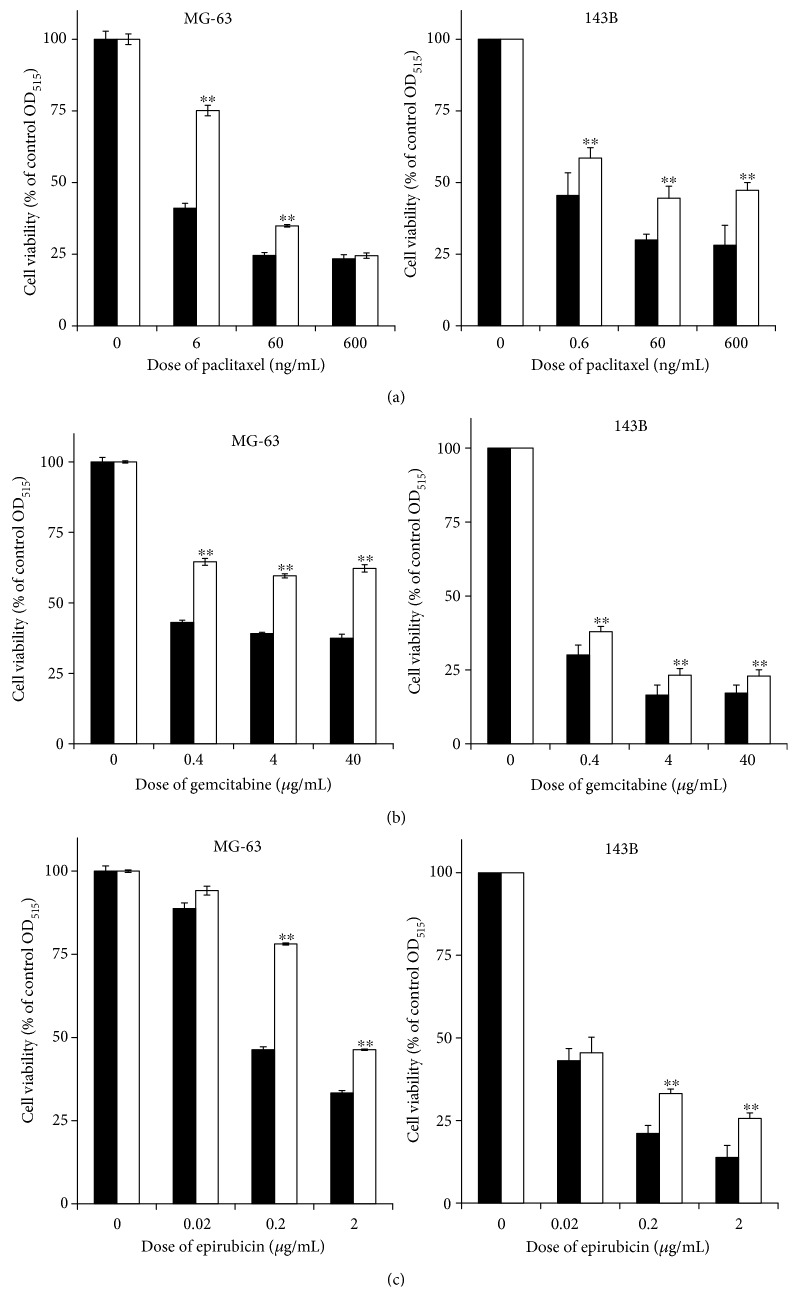
The role of GRP94 in chemotherapeutic resistance. The scrambled control and GRP94-KD OS (MG63 and 143B) cells were treated with different doses of (a) paclitaxel (0–600 ng/mL), (b) gemcitabine (0–40 *μ*g/mL), or (c) epirubicin (0–2 *μ*g/mL). The results reported were obtained from at least three independently repeated experiments (^∗∗^*p* < 0.01).

Scrambled control and GRP94-KD cells were treated with different doses of paclitaxel (0–600 ng/mL), gemcitabine (0–40 *μ*g/mL), or epirubicin (0–2 *μ*g/mL), and the 50% growth inhibition (IC_50_) doses in the GRP94-KD and scrambled control MG63 and 143B cells were determined to identify the role in mediating the cellular response to chemotherapy. As shown in Figure 2, GRP94-KD cells exhibited increased IC_50_ values for paclitaxel, gemcitabine, and epirubicin compared with scrambled control cells. Based on these results, GRP94 silencing may increase the resistance of OS cells to paclitaxel, gemcitabine, and epirubicin treatments.

### 3.3. Knockdown of GRP94 Inhibited Chemotherapy-Induced Apoptosis in OS Cells

**Figure 3:**
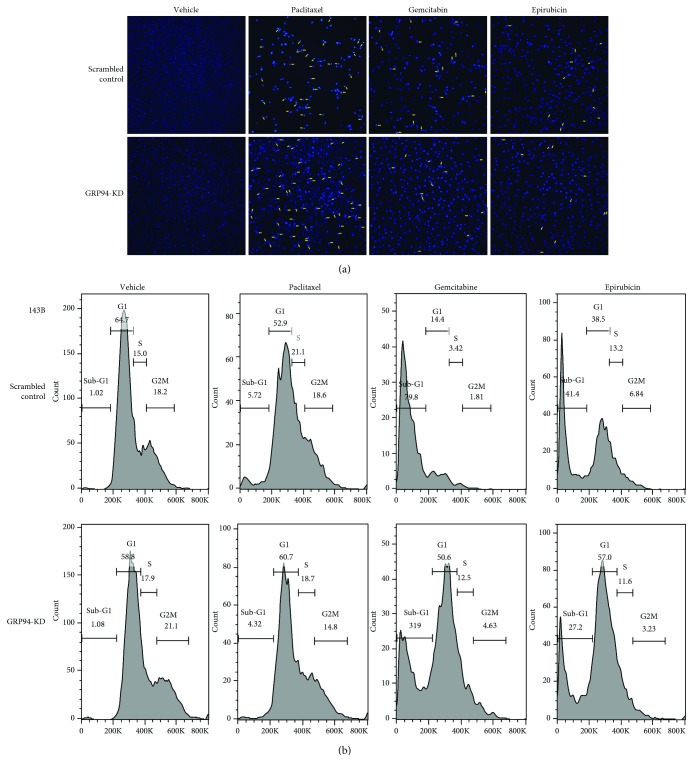
GRP94-KD inhibited chemotherapy-induced apoptosis in OS cells. (a) Representative DAPI staining of MG63 cells. Scrambled control and GRP94-KD MG63 cells were incubated with paclitaxel (6 ng/mL), gemcitabine (4 *μ*g/mL), or epirubicin (2 *μ*g/mL) for 48 hr and then stained with DAPI. Apoptotic cells were imaged using a fluorescence microscope. (b) The populations of scrambled control and GRP94-KD 143B cells in different phases of the cell cycle and sub-G_1_ populations after chemotherapy treatment. The flow cytometry analysis of the cell cycle was performed by staining DNA with PI. The results reported were obtained from at least three independently repeated experiments.

DAPI staining was performed to further explore how GRP94 expression influences the cytotoxic effects of paclitaxel, gemcitabine, and epirubicin on MG63 cells and 143B cells. Chemotherapy-induced apoptosis in a greater number of scrambled control cells than in GRP94-KD MG63 cells, as shown by the DAPI staining (Figure 3(a)). In scrambled control cells, it shows lower cell densities and higher apoptotic cell population upon exposure to paclitaxel, gemcitabine, and epirubicin. We further confirmed the cytotoxic effects of paclitaxel, gemcitabine, and epirubicin on scrambled control and GRP94-KD OS 143B cells using PI staining. As shown in Figure 3(b), the sub-G_1_ population of scrambled control 143B cells was increased after paclitaxel, gemcitabine, and epirubicin exposure. However, the sub-G_1_ population of GRP94-KD-treated cells was reduced compared with that of scrambled control-treated cells. Thus, GRP94 silencing may reduce the sensitivity of OS cells to paclitaxel, gemcitabine, and epirubicin treatments.

### 3.4. Determination of the Activity and Expression Levels of Caspases 3/7

**Figure 4:**
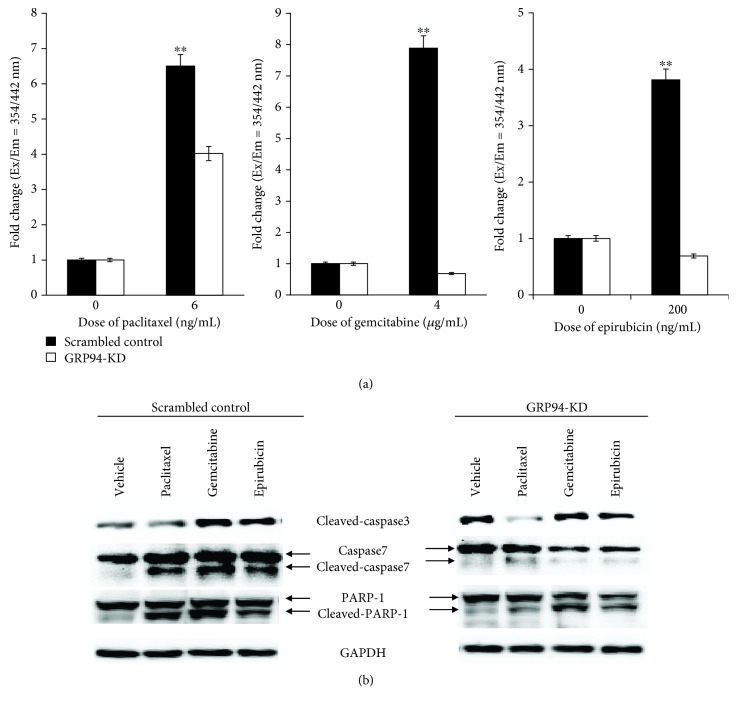
GRP94-KD decreased the chemotherapy-induced caspase 3/7 levels. (a) Caspase 3/7 activities in MG63 cells treated with paclitaxel, gemcitabine, and epirubicin. Fluorescence intensity indicated the level of activated caspases 3/7 in the scrambled control and GRP94-KD MG63 cells after exposure to chemotherapeutic drugs. (b) The levels of cleaved caspase 3, caspase 7, cleaved caspase 7, cleaved PARP-1, and GAPDH in scrambled control and GRP94-KD 143B cells were confirmed by Western blotting. The results reported were obtained from at least three independently repeated experiments (^∗∗^*p* < 0.01).

The activities of caspase 3 and caspase 7, which are executor caspases that act together to facilitate apoptosis, were determined in MG63 cells using a fluorometric assay to further confirm these results. Briefly, in this assay, the hydrolysis of a specific substrate results in the generation of a fluorescent molecule, and thus, the fluorescence intensity reflects the activities of caspases 3/7. Paclitaxel, gemcitabine, and epirubicin treatment dramatically increased caspase 3/7 activities in scrambled control cells compared to GRP94-KD cells (Figure 4(a)). Next, we confirmed the levels of the caspase 3 and caspase 7 proteins by Western blotting. The levels of cleaved caspases 3 and 7 were dramatically increased in OS cells treated with paclitaxel, gemcitabine, and epirubicin (Figure 4(b)). The levels of cleaved caspase 3, caspase 7, and PARP were increased to a greater extent in scrambled control-treated cells than in GRP94-KD cells. Based on these data, GRP94 silencing induces chemotherapy resistance in OS cells due to the suppression of the caspase-mediated mitochondrial cell death pathway.

## 4. Discussion

OS is a highly aggressive malignant bone tumor, with approximately 20% of all patients presenting with metastasis at the initial visit [19]. Primary OS has been shown to be resistant to high-dose chemotherapy, and its 5-year overall survival rate has plateaued at 60–70% in the past two decades [20–22]. Therefore, the identification of prognostic or therapeutic biomarkers that may enhance the therapeutic response of OS and improve the management approaches for this disease is critically needed. Higher levels of GRP94 are correlated with poor disease outcomes in different cancers [23, 24], but there is limited information of the roles of GRP94 in therapeutic response in OS. The current study has demonstrated that GRP94 silencing does not influence the proliferation or migration, but causes a reduction in colony formation in both MG63 cells and 143B cells. The finding that GRP94 may not be involved in the mechanism regulating the growth and migration is unique to OS compared with the findings from other cancers. In addition, GRP94-KD reduced the colony formation ability, indicating that GRP94 may be correlated with the malignant features of OS. GRP94-KD also decreased cells' sensitivity to chemotherapeutic drugs (paclitaxel, gemcitabine, and epirubicin).

GRP94 is well known for its therapeutic and prognostic roles in cancer. GRP94 is induced as a defense mechanism for the survival of cancer cells exposed to stressful conditions [10]. It is elevated as a response to the inhibition of glycosylation, Ca^2+^ pool depletion, and malfolded proteins and is regulated through antiapoptotic (BCL-2) target proteins [25, 26]. ER stress may induce apoptotic signaling pathways as cells mount the unfolded protein response (UPR) as a self-protective mechanism for ER function disruption [27, 28]. This leads to the accumulation of different unfolded or misfolded proteins in ER [28]. GRP78 and GRP94 expression are hallmarks of ER stress and UPR [29, 30]. Caspase-mediated apoptosis is said to be an important mechanism which regulates tumor progression [26]. McCormick et al. observed that mouse lymphoma cells that fail to mount GRP94 stress response are more susceptible to the inhibitor of Ca^2+^ uptake into the ER, thapsigargin (TG) [31]; interestingly, the inhibition of GRP94 stress response did not enhance the cytotoxicity of the inhibitor of *N*-linked glycosylation, tunicamycin (TN) [32]. This suggests that two pathways may be involved in the regulation of GRP94: glycosylation inhibition mediated and the one mediated by Ca^2+^ [31], where GRP94 expression promotes radio-chemotolerance in cancer cells during the maintenance of cellular Ca^2+^ homeostasis when combating ER stress, after going through cleavage by calpain, simultaneously preventing apoptosis [9].

Fu et al. observed a similar phenomenon in multiple myeloma (MM) where cells expressing low GRP94 and GRP78 were resistant to bortezomib (BTZ). In this study, inducing ER stress with tunicamycin reversed drug resistance of MM cells by inhibiting the PI3K/Akt/mTOR signaling pathway [33]. The same phenomenon has also been observed in ovarian, breast, esophageal, and lung cancer cells treated with different agents and radiotherapy [34–38]. Our previous study also identified a novel pathway by which GRP94 regulates resistance, whereby GRP94 knockdown reduced the sensitivity to taxanes by suppressing the caspase-mediated mitochondrial cell death pathway and by altering the activation of apoptosis and associated proteins [39]. Agreeing with these findings, we propose that GRP94-KD OS cells were resistant to chemotherapy because of their failure to respond to ER stress, which lead to reduced apoptosis and therefore treatment response.

In addition to the above discussion, knockdown of GRP94 leads to AKT activation and the expansion of hematopoietic stem cells (HSCs), which correspond with the loss of surface expression of integrin *β*4 and HSC niche attachment [40, 41]. The liver-specific knockout of GRP94 in mice disrupts cell adhesion, activates liver progenitor cells, and accelerates liver tumorigenesis [42]. These observations may partly explain our finding that GRP94-KD impaired anchorage-dependent colony formation. Therefore, GRP94 protects cells from the host defense systems and promotes tumor progression and therapeutic response through its pro-proliferation and antiapoptotic functions [43].

Although the mechanisms are not fully explored in the present study, it has provided important insights into the role of GRP94 in OS, which are crucial for the management of OS and the development of novel drug targets.

## Data Availability

The data used to support the findings of this study are available from the corresponding author upon request.

## References

[1] Luetke A., Meyers P. A., Lewis I., Juergens H. (2014). Osteosarcoma treatment—where do we stand? A state of the art review. *Cancer Treatment Reviews*.

[2] Bielack S. S., Kempf-Bielack B., Delling G. (2002). Prognostic factors in high-grade osteosarcoma of the extremities or trunk: an analysis of 1,702 patients treated on neoadjuvant cooperative osteosarcoma study group protocols. *Journal of Clinical Oncology*.

[3] Isakoff M. S., Bielack S. S., Meltzer P., Gorlick R. (2015). Osteosarcoma: current treatment and a collaborative pathway to success. *Journal of Clinical Oncology*.

[4] Chang J. T.-C., Chan S. H., Lin C. Y. (2007). Differentially expressed genes in radioresistant nasopharyngeal cancer cells: gp96 and GDF15. *Molecular Cancer Therapeutics*.

[5] Bando Y., Katayama T., Kasai K., Taniguchi M., Tamatani M., Tohyama M. (2003). GRP94 (94 kDa glucose-regulated protein) suppresses ischemic neuronal cell death against ischemia/reperfusion injury. *European Journal of Neuroscience*.

[6] di Michele M., Marcone S., Cicchillitti L. (2010). Glycoproteomics of paclitaxel resistance in human epithelial ovarian cancer cell lines: towards the identification of putative biomarkers. *Journal of Proteomics*.

[7] Lee A. S. (2001). The glucose-regulated proteins: stress induction and clinical applications. *Trends in Biochemical Sciences*.

[8] Lee A. S. (2014). Glucose-regulated proteins in cancer: molecular mechanisms and therapeutic potential. *Nature Reviews Cancer*.

[9] Reddy R. K., Lu J., Lee A. S. (1999). The endoplasmic reticulum chaperone glycoprotein GRP94 with Ca^2+^-binding and antiapoptotic properties is a novel proteolytic target of calpain during etoposide-induced apoptosis. *Journal of Biological Chemistry*.

[10] Fu Y., Lee A. S. (2006). Glucose regulated proteins in cancer progression, drug resistance and immunotherapy. *Cancer Biology & Therapy*.

[11] Kim Y. S., Alarcon S. V., Lee S. (2009). Update on Hsp90 inhibitors in clinical trial. *Current Topics in Medicinal Chemistry*.

[12] Biamonte M. A., van de Water R., Arndt J. W., Scannevin R. H., Perret D., Lee W.-C. (2010). Heat shock protein 90: inhibitors in clinical trials. *Journal of Medicinal Chemistry*.

[13] Duerfeldt A. S., Peterson L. B., Maynard J. C. (2012). Development of a Grp94 inhibitor. *Journal of the American Chemical Society*.

[14] Huang C. Y., Batzorig U., Cheng W. L. (2016). Glucose-regulated protein 94 mediates cancer progression via AKT and eNOS in hepatocellular carcinoma. *Tumour Biology*.

[15] Sowinski S., Jolly C., Berninghausen O. (2008). Membrane nanotubes physically connect T cells over long distances presenting a novel route for HIV-1 transmission. *Nature Cell Biology*.

[16] Wang S.-K., Liang P. H., Astronomo R. D. (2008). Targeting the carbohydrates on HIV-1: interaction of oligomannose dendrons with human monoclonal antibody 2G12 and DC-SIGN. *Proceedings of the National Academy of Sciences of the United States of America*.

[17] Liang H. H., Huang C. Y., Chou C. W. (2018). Heat shock protein 27 influences the anti-cancer effect of curcumin in colon cancer cells through ROS production and autophagy activation. *Life Sciences*.

[18] Hung C.-S., Liu H.-H., Liu J.-J. (2013). MicroRNA-200a and -200b mediated hepatocellular carcinoma cell migration through the epithelial to mesenchymal transition markers. *Annals of Surgical Oncology*.

[19] Hughes D. P. M. (2009). Strategies for the targeted delivery of therapeutics for osteosarcoma. *Expert Opinion on Drug Delivery*.

[20] Fuchs N., Bielack S. S., Epler D. (1998). Long-term results of the co-operative German-Austrian-Swiss osteosarcoma study group’s protocol COSS-86 of intensive multidrug chemotherapy and surgery for osteosarcoma of the limbs. *Annals of Oncology*.

[21] Philip T., Iliescu C., Demaille M. C. (1999). High-dose methotrexate and HELP [Holoxan (ifosfamide), Eldesine (vindesine), platinum]—doxorubicin in non-metastatic osteosarcoma of the extremity: a French multicentre pilot study. *Annals of Oncology*.

[22] Ferrari S., Smeland S., Mercuri M. (2005). Neoadjuvant chemotherapy with high-dose ifosfamide, high-dose methotrexate, cisplatin, and doxorubicin for patients with localized osteosarcoma of the extremity: a joint study by the Italian and Scandinavian Sarcoma Groups. *Journal of Clinical Oncology*.

[23] Martínez-Aranda A., Hernández V., Guney E. (2015). FN14 and GRP94 expression are prognostic/predictive biomarkers of brain metastasis outcome that open up new therapeutic strategies. *Oncotarget*.

[24] Chen G. N., Ma Y., Yang Z. L. (2010). Expression of GRP78 and GRP94 in the liver tissues and their clinicopathological significance in children with hepatoblastoma. *Zhongguo Dang Dai Er Ke Za Zhi*.

[25] Insel P. A., Zhang L., Murray F., Yokouchi H., Zambon A. C. (2012). Cyclic AMP is both a pro-apoptotic and anti-apoptotic second messenger. *Acta Physiologica*.

[26] Spampanato C., de Maria S., Sarnataro M. (2012). Simvastatin inhibits cancer cell growth by inducing apoptosis correlated to activation of Bax and down-regulation of BCL-2 gene expression. *International Journal of Oncology*.

[27] Zhang R., Piao M. J., Kim K. C. (2012). Endoplasmic reticulum stress signaling is involved in silver nanoparticles-induced apoptosis. *The International Journal of Biochemistry & Cell Biology*.

[28] Cnop M., Foufelle F., Velloso L. A. (2012). Endoplasmic reticulum stress, obesity and diabetes. *Trends in Molecular Medicine*.

[29] Carlisle R. E., Heffernan A., Brimble E. (2012). TDAG51 mediates epithelial-to-mesenchymal transition in human proximal tubular epithelium. *American Journal of Physiology-Renal Physiology*.

[30] Du L., He F., Kuang L., Tang W., Li Y., Chen D. (2017). eNOS/iNOS and endoplasmic reticulum stress-induced apoptosis in the placentas of patients with preeclampsia. *Journal of Human Hypertension*.

[31] McCormick T. S., McColl K. S., Distelhorst C. W. (1997). Mouse lymphoma cells destined to undergo apoptosis in response to thapsigargin treatment fail to generate a calcium-mediated *grp78/grp9*4 stress response. *Journal of Biological Chemistry*.

[32] Little E., Lee A. S. (1995). Generation of a mammalian cell line deficient in glucose-regulated protein stress induction through targeted ribozyme driven by a stress-inducible promoter. *Journal of Biological Chemistry*.

[33] Fu Y. F., Liu X., Gao M., Zhang Y. N., Liu J. (2017). Endoplasmic reticulum stress induces autophagy and apoptosis while inhibiting proliferation and drug resistance in multiple myeloma through the PI3K/Akt/mTOR signaling pathway. *Oncotarget*.

[34] Yu X.-S., du J., Fan Y.-J. (2016). Activation of endoplasmic reticulum stress promotes autophagy and apoptosis and reverses chemoresistance of human small cell lung cancer cells by inhibiting the PI3K/AKT/mTOR signaling pathway. *Oncotarget*.

[35] Zhou F., Li Y. H., Wang J. J., Pan J., Lu H. (2017). Endoplasmic reticulum stress could induce autophagy and apoptosis and enhance chemotherapy sensitivity in human esophageal cancer EC9706 cells by mediating PI3K/Akt/mTOR signaling pathway. *Tumour Biology*.

[36] Pang X. L., He G., Liu Y. B., Wang Y., Zhang B. (2013). Endoplasmic reticulum stress sensitizes human esophageal cancer cell to radiation. *World Journal of Gastroenterology*.

[37] Zhong J. T., Yu J., Wang H. J. (2017). Effects of endoplasmic reticulum stress on the autophagy, apoptosis, and chemotherapy resistance of human breast cancer cells by regulating the PI3K/AKT/mTOR signaling pathway. *Tumour Biology*.

[38] Hu J. L., Hu X. L., Guo A. Y., Wang C. J., Wen Y. Y., Cang S. D. (2017). Endoplasmic reticulum stress promotes autophagy and apoptosis and reverses chemoresistance in human ovarian cancer cells. *Oncotarget*.

[39] Tai C. J., Wang J. W., Su H. Y. (2014). Glucose-regulated protein 94 modulates the therapeutic efficacy to taxane in cervical cancer cells. *Tumour Biology*.

[40] Luo B., Lam B. S., Lee S. H. (2011). The endoplasmic reticulum chaperone protein GRP94 is required for maintaining hematopoietic stem cell interactions with the adult bone marrow niche. *PLoS One*.

[41] Luo B., Tseng C. C., Adams G. B., Lee A. S. (2013). Deficiency of GRP94 in the hematopoietic system alters proliferation regulators in hematopoietic stem cells. *Stem Cells and Development*.

[42] Chen W. T., Ha D., Kanel G., Lee A. S. (2014). Targeted deletion of ER chaperone GRP94 in the liver results in injury, repopulation of GRP94-positive hepatocytes, and spontaneous hepatocellular carcinoma development in aged mice. *Neoplasia*.

[43] Chen W. T., Tseng C. C., Pfaffenbach K. (2014). Liver-specific knockout of GRP94 in mice disrupts cell adhesion, activates liver progenitor cells, and accelerates liver tumorigenesis. *Hepatology*.

